# Effectiveness of a combination of cognitive behavioral therapy and task-oriented balance training in reducing the fear of falling in patients with chronic stroke: study protocol for a randomized controlled trial

**DOI:** 10.1186/s13063-018-2549-z

**Published:** 2018-03-07

**Authors:** Tai-Wa Liu, Gabriel Y. F. Ng, Shamay S. M. Ng

**Affiliations:** 1 Department of Rehabilitation Sciences, The Hong Kong Polytechnic University, Hung Hom, Hong Kong, Special Administrative Region of China; 2 Division of Nursing and Health Studies, The Open University of Hong Kong, Ho Man Tin, Hong Kong, Special Administrative Region of China

**Keywords:** Stroke rehabilitation, Cognitive behavioral therapy, Fear of falling, Subjective balance confidence, Balance self-efficacy, Fall risk

## Abstract

**Background:**

The consequences of falls are devastating for patients with stroke. Balance problems and fear of falling are two major challenges, and recent systematic reviews have revealed that habitual physical exercise training alone cannot reduce the occurrence of falls in stroke survivors. However, recent trials with community-dwelling healthy older adults yielded the promising result that interventions with a cognitive behavioral therapy (CBT) component can simultaneously promote balance and reduce the fear of falling. Therefore, the aim of the proposed clinical trial is to evaluate the effectiveness of a combination of CBT and task-oriented balance training (TOBT) in promoting subjective balance confidence, and thereby reducing fear-avoidance behavior, improving balance ability, reducing fall risk, and promoting independent living, community reintegration, and health-related quality of life of patients with stroke.

**Methods:**

The study will constitute a placebo-controlled single-blind parallel-group randomized controlled trial in which patients are assessed immediately, at 3 months, and at 12 months. The selected participants will be randomly allocated into one of two parallel groups (the experimental group and the control group) with a 1:1 ratio. Both groups will receive 45 min of TOBT twice per week for 8 weeks. In addition, the experimental group will receive a 45-min CBT-based group intervention, and the control group will receive 45 min of general health education (GHE) twice per week for 8 weeks. The primary outcome measure is subjective balance confidence. The secondary outcome measures are fear-avoidance behavior, balance ability, fall risk, level of activities of daily living, community reintegration, and health-related quality of life.

**Discussion:**

The proposed clinical trial will compare the effectiveness of CBT combined with TOBT and GHE combined with TOBT in promoting subjective balance confidence among chronic stroke patients.

We hope our results will provide evidence of a safe, cost-effective, and readily transferrable therapeutic approach to clinical practice that reduces fear-avoidance behavior, improves balance ability, reduces fall risk, promotes independence and community reintegration, and enhances health-related quality of life.

**Trial registration:**

ClinicalTrials.gov, NCT02937532. Registered on 17 October 2016.

## Background

Fear of falling (FoF) is one of the most common post-stroke complications, and is widely acknowledged as part of a vicious circle [1] leading to actual falls [2]. It is a debilitating post-fall syndrome stemming from low balance self-efficacy and the fearful anticipation of falling [3, 4]. The reported prevalence of FoF varies between post-stroke stages, ranging from 54% before discharge [5] from an acute unit to 44% at 6 months after stroke [6] and 58% among community-dwelling patients with stroke [7]. If no action is taken, FoF spirals into a loss of physical function, dependency on others for assistance with activities of daily living (ADL), restrictions on daily activities [4], and a higher fall rate, [8] eventually compromising community integration [9].

Two recent systematic reviews synthesized the findings of interventions targeting FoF. Bula et al.’s [10] review of 46 randomized controlled trials (RCTs) with 6794 community-dwelling elderly persons revealed that the majority of the reviewed studies (*n* = 38) focused on fall prevention and balance improvement, with FoF regarded as a secondary outcome. In the eight studies directly addressing the fear of fearing, the use of physiological interventions such as tai chi [11]; strengthening, balance and walking exercises [12]; psychological interventions such as cognitive behavioral therapy (CBT) [13, 14]; and guided relaxation and exercise imagery [15] was reported to help reduce FoF among community-dwelling older people.

In another systematic review, Tang et al. [16] examined 19 clinical trials addressing FoF among people with stroke. Despite its significant influence on stroke rehabilitation, FoF was regarded only as a secondary target in the studies reviewed. Tang’s [16] meta-analysis of 15 clinical trials with 627 participants revealed that intensive exercise-based physiological interventions, such as gait training [17–20], exergaming [21], yoga [22], and a combination of fitness, mobility and functional exercises [23, 24], can reduce FoF with a medium effect size (standardized mean difference 0.44; 95% confidence interval (0.11–0.77); *p* = 0.009). No improvements were noted in the four reviewed studies using psychological interventions (motor imagery) [25–28], and no retention effect was noted in the studies with a follow-up assessment. However, the effectiveness of CBT as a psychological intervention in reducing the FoF of stroke patients has not been examined.

CBT is a psychotherapeutic approach that redirects negative cognitive, emotional, or behavioral responses to help people develop coping mechanisms and self-confidence [29]. For example, people with FoF originating from impaired balance self-efficacy can use CBT to change their self-defeating beliefs, improve their balance self-efficacy and replace their unrealistic anticipation of falls and magnified FoF consequences with a realistic, positive perspective on falls, in turn reducing their fear avoidance.

As summarized by Bula et al. [10] and Tang et al. [16], studies have shown that physical exercise can reduce FoF in older people and people with stroke as either a primary or a secondary outcome. As psychological interventions offer another possible means of reducing FoF, we aim to examine the effectiveness of a combination of CBT and task-oriented balance training (TOBT) in reducing the FoF of people with stroke. TOBT will be used in the proposed study because it targets stroke-specific impairments and has been clinically proven to improve the balance performance of people with stroke [30, 31]. The inclusion of CBT in our treatment arm is based on our hypothesis that CBT is an adjunct therapy capable of optimizing the treatment effects of exercise in reducing FoF. It is expected to tackle FoF directly through the promotion of balance self-efficacy, and its indirect effects will be mediated by repeated exercise and reduced fear-avoidance behavior, further enhancing balance performance and ADL, and thereby improving community integration. The combined effects of CBT and TOBT in reducing FoF are expected to improve patients’ balance, reduce their risk of falling, increase their independence, and thereby promote their community integration. Indeed, in Huang et al.’s [32] recent RCT with elderly persons, CBT with an exercise intervention (*n* = 27) performed better than either CBT alone (*n* = 27) or treatment as usual (*n* = 26) in reducing FoF and depression and enhancing mobility and muscle strength, with retention effects observed up to 5 months later. Therefore, the proposed study aims to determine whether combining CBT with TOBT augments the latter’s positive treatment effects on FoF, and thus fear-avoidance behavior, balance ability, fall risk, independent living, enhancing community integration, and health-related quality of life among community-dwelling seniors with stroke.

To develop an intervention for clinical use, a protocol is necessary to ensure the consistency of implementation and ease of replication. Therefore, the objective of this paper is to report the details of a protocol for combining CBT and TOBT to reduce FoF among people with stroke.

## Methods

### Trial design

The proposed study will be a placebo-controlled single-blind parallel-group RCT with a 12-month follow-up, conducted with community-dwelling chronic stroke survivors with FoF at a university-based rehabilitation center. The findings of the trial will be reported in accordance with the Consolidated Standards of Reporting Statement [33].

### Choice of comparator

A placebo control intervention, general health education (GHE), will be provided for the control group to help measure the effects of CBT alone. To rule out potential placebo effects such as attention from therapists and knowledge of treatment conditions, the GHE program will provide no information related to subjective balance confidence, activity avoidance, falls, or physical activity, but only information related to general health issues such as healthy food choices and foot care.

### Null hypothesis

The null hypothesis will be that the efficacy of CBT combined with TOBT does not differ significantly from that of GHE combined with TOBT in promoting balance self-efficacy, thus reducing fear-avoidance behavior, enhancing balance ability, reducing fall risk, and improving community reintegration and health-related quality of life for people with stroke.

### Participants

Prospective participants will be required to meet the following inclusion criteria: (i) aged between 55 and 85, (ii) diagnosed with a first unilateral ischemic brain injury or intracerebral hemorrhage by magnetic resonance imaging or computed tomography within 1–6 years post-stroke, (iii) discharged from all rehabilitation services at least 6 months before the program, (iv) able to walk independently for at least 10 m with or without an assistive device, (v) showing low balance self-efficacy [scoring less than 80 on the Chinese version of the Activities-specific Balance Confidence (ABC-C) Scale] [34], (vi) scoring higher than 7 out of 10 on the Chinese version of the Abbreviated Mental Test [35], and (vii) able to follow instructions and provide written informed consent.

Individuals will be excluded if they have any additional medical, cardiovascular, orthopedic, psychiatric, or psychological conditions that will hinder proper treatment or assessment, if they present with receptive dysphasia or significant lower limb peripheral neuropathy, or if they are involved in drug studies or other clinical trials.

### Therapists and research personnel

Two research assistants with at least 2 years of research experience in physical exercise training will be the assessors of this study. They will be given a 1-day training session on obtaining outcome measurements by an experienced physiotherapist before the study. Training will be provided in both the theory and practice of using the outcome measures. All of the assessors will rehearse the outcome measures with the research team personnel to standardize the assessment. To establish the interrater reliability, the two assessors will rate five participants and then review for discrepancies, if any.

The two TOBT therapists will have been trained by an experienced physiotherapist and have at least 2 years of post-qualification experience as therapists in physical exercise training. They will be provided with written progression guidelines (Table 1). A regular review of training records and spot observations will be conducted by the experienced physiotherapist to enhance adherence to the written progression guidelines. The CBT therapists will be three psychiatric nurses who have qualified as cognitive therapists. They will all have at least 5 years of post-qualification experience with applying CBT clinically. A treatment manual and materials have already been developed with reference to Tennstedt et al.’s [13] and Zijlstra et al.’s [14] research on FoF as experienced by community-dwelling older adults and reviewed by the three certified cognitive therapists involved in the study. To ensure treatment integrity, the CBT intervention has already been piloted and audiotaped. Each CBT therapist evaluated the pilot sessions to assess their compliance with the treatment manual, the achievement of session goals, and the use of CBT techniques. The GHE intervention will be delivered by two research assistants not involved in the assessment or any other part of the intervention, using audio-visual aids and materials that have already been developed.

**Table 1:** Progression criteria for task-oriented balance training

Exercise	Progression criteria	Method of progression
Stepping up and down	Able to complete 50 times	Starting with a 2-in.-high wooden step, then progressing to 4- and 6-in.-high wooden steps after the progression criteria have been met
Heel-raising exercises	Able to complete 25 times with at least 5 s held on each repetition	Starting with a 2-in.-high wooden step, then progressing to 4- and 6-in.-high wooden ramp after the progression criteria have been met
Semi-squatting	Able to maintain knee flexion angle of 30 degrees without obvious shaking	Starting with a 3-min rest interval midway through the trial, which is subsequently reduced to 2 min, 1 min, and 0 min
Standing on duraDisc	Able to stand without external assistance for at least 1 min (holding handrail or supported by another)	Decrease the base of support
Walking across obstacles	Able to complete the task within a pre-set duration (20 s at the beginning) without knocking down the obstacles	Shorten the pre-set duration and increase number of obstacles

### Procedure

Participants will be recruited from a local self-help group for people with stroke through poster advertisements. On receiving telephone calls from interested parties, our recruitment research assistant will perform an initial eligibility screening and offer appointments to gain written informed consent and complete a baseline assessment.

All of the potential participants will meet individually in the study venue to enable the researchers to explain the details of the study, such as its aims, benefits, risks, and confidentiality, and then check the applicants’ eligibility against the inclusion and exclusion criteria. If individuals are both interested in joining and eligible to join the clinical trial, written informed consent will be obtained before the baseline assessment is conducted. Questionnaires relating to sociodemographic characteristics, variables of interest, and physical and functional performance will be completed on the same day.

### Measurements

**Fig. 1:**
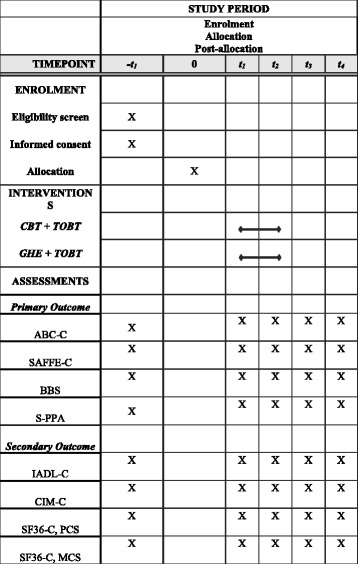
Schedule of enrollment, interventions, and assessments. ABC-C Activities-specific Balance Confidence Scale (Chinese version), BBS Berg Balance Scale, CBT cognitive behavioral therapy, CIM-C Community Integration Measure (Chinese version), GHE general health education, IADL-C Lawton Instrumental Activities of Daily Living (Chinese version), SAFFE-C Survey of Activities and Fear of Falling in the Elderly (Chinese version), SF36-C MCS mental component of the Chinese version of the Short Form General Health Questionnaire, SF36-C PCS physical component of the Chinese version of the Short Form General Health Questionnaire, S-PPA Short-form Physiological Profile Assessment, TOBT task-oriented balance training

All of the participants will be required to undergo five sets of measurements (Fig. 1): (i) before assessment (baseline treatment), (ii) after eight sessions of treatment (midway through treatment), (iii) after 16 sessions of treatment (end of treatment), (iv) 12 weeks after treatment (follow-up), and (v) 12 months after treatment (follow-up). All of the assessment procedures will be performed by a research assistant blind to the group allocation and not previously involved in the delivery of the interventions.

### Randomization and blinding

**Fig. 2:**
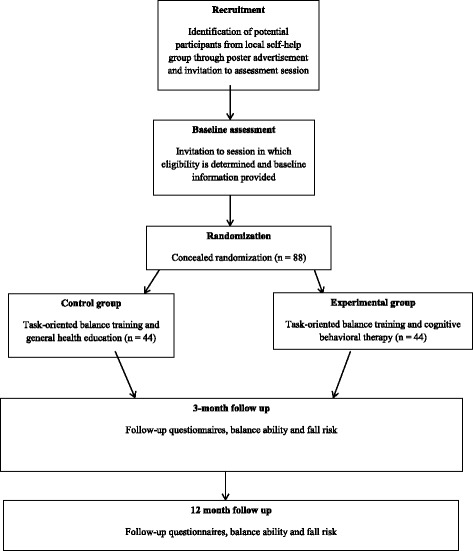
Flow diagram for clinical trial

Figure 2 presents an overview of the study. After explaining the study’s objectives and obtaining written informed consent, a research assistant will perform a baseline assessment for all of the outcome measures. An offsite volunteer not involved in the recruitment, intervention, or data collection will randomly allocate the participants to either the experimental group or control group in a 1:1 ratio, using the computer program Minimise [36]. The randomization will be stratified based on age (55–70 years or 71–85 years), gender (male or female), and level of subjective balance confidence based on ABC-C scores (< 50 or 50–80) [37]. The participants will be informed of the results of the group allocation and their resulting training schedule and venue by centralized telephone calls from an offsite volunteer to ensure concealed randomization.

To maintain assessor blinding, the assessment, data entry, and data analysis will all be performed by another full-time research assistant blind to group allocation and not involved in delivering the interventions. The intervention and assessment will be physically separate, performed at different sites. The subjects will be reminded not to disclose any information on their intervention groups to the assessors. However, it will be impossible to blind the therapists and participants to the group allocation.

All of the participants will be asked to undertake 16 sessions of training over an 8-week period. The participants in both groups will undergo 45-min sessions of TOBT in groups of three to five. TOBT is a rehabilitation strategy designed to improve muscle strength in lower limbs and to correct for balance deficits on the paretic side of patients with stroke [30, 38, 39]. Improved strength and balance are gained through the repetition of task-specific functional movements.

The TOBT intervention will consist of five exercises targeting muscle strength in the lower limbs and walking performance: (i) stepping up and down in different directions to strengthen the affected leg muscles and to increase control over shifts in the center of gravity, (ii) heel-raising exercises to strengthen the ankle plantar flexors, (iii) semi-squatting to improve lower limb strength and proprioception in the knees and ankles, (iv) standing on a duraDisc to promote static balance, and (v) walking across a surface covered with obstacles to improve dynamic walking balance.

Based on our practical experience of using TOBT in previous studies of patients with chronic stroke [30, 40], the proposed frequency and intensity of treatment will be effective and tolerable, providing sufficient stimulation to enhance motor recovery in patients with stroke. In the TOBT sessions, the participants will take turns in carrying out one of the TOBT exercises for 8 min, followed by a 1-min rest interval, until the five TOBT exercises have been completed. All the TOBT sessions will be held in the morning, and then the participants attend either the CBT or GHE session on the same day in the afternoon after a 2-hour lunch break.

#### Experimental group

The experimental group will receive twice weekly CBT sessions for 8 weeks, lasting for 45 min per session, in groups of three to five. The CBT sessions will be focused on eliminating cognitive and behavioral factors known to generate and aggravate impaired subjective balance confidence and fear-avoidance behavior. The aim will be to increase the self-perception of efficacy regarding falls and the sense of control over falling, to decrease the perception of risk, and to help the participants adopt realistic expectations of the consequences of falls. Each week will have a specific theme in the CBT protocol. The main themes and content are summarized in Table 2.

**Table 2:** Weekly themes and main content of CBT sessions

Week	Weekly theme
1	Introduction and briefing on the aims of the rehabilitation program • Introduction to group • Introduction of the concept of self-efficacy • Information on post-stroke balance self-efficacy and rehabilitation
2	Understanding the relationships between thoughts, emotions, and behavior • Introduction to the CBT model • Understanding fear, fear of falling, fall risks, and actual falls • Understanding the automatic thoughts and emotional and behavioral reflection associated with fear of falling
3	Exploring thoughts and maladaptive responses • Identifying maladaptive thoughts leading to physical inactivity • Adapting realistic views of fall risk and the consequences of falls • Recognizing risky behavior • Overcoming barriers to physical activity
4	Exploring adaptive thoughts and behavioral responses • Fall prevention strategies and safety issues • Recognizing and minimizing fall risk
5	Implementing and reviewing behavioral changes related to ADL • Setting personal goals for ADL • Planning to achieve personal goals in small stages • Recognizing potential hazards and planning for safety
6	Implementing and reviewing behavioral changes related to social activities • Setting personal goals for social activities • Planning to achieve personal goals in small stages • Recognizing potential hazards and planning for safety
7	Reviewing and advancing individual therapeutic goals • Reviewing personal goals for ADL and social activities • Establishing a regular exercise plan
8	Consolidating the experiences of the rehabilitation program • Sharing attitudes and experiences of fear of falling before and after the group • Sharing experiences of applying cognitive-restructuring skills • Establishing the long-term personal goals of regular exercise, ADL independence, and social engagement

*ADL* activities of daily living, *CBT* cognitive behavioral therapy

Weeks 1 and 2 of the CBT sessions will focus on introducing the CBT framework and showing how self-perceived capability and maladaptive thoughts can influence behavioral performance. From week 3 to week 8, two major techniques, cognitive restructuring and behavioral modification, will be used in the CBT sessions to achieve participants’ personal goals. Cognitive restructuring attains thought alteration in the following four steps: (1) identification of automatic thoughts, (2) examination of cognitive distortion originating from automatic thoughts, (3) disputing the cognitive distortion and automatic thoughts, and (4) developing adaptive beliefs. In the proposed study, these four steps will be undertaken in the form of CBT homework assignments and sharing and discussion during CBT group sessions.

After effecting cognitive restructuring, the CBT group sessions will target behavioral modification, another crucial component of the intervention. The participants will be equipped to identify potential risks and develop behavioral strategies to prepare them to increase their activity levels safely. The CBT sessions will also serve as a platform for vicarious learning, social persuasion, and social modeling for the participants through group discussion and observing the success of others. In addition, mastery experiences can be gained through the successful application of CBT in daily situations. Indeed, according to Bandura [41], this is the major source of self-efficacy. Therefore, the use of cognitive-restructuring and behavioral-modification techniques is expected to enhance the participants’ subjective balance confidence and reduce their fear-related avoidance behavior.

#### Control group

The control group will attend 16 health talks (two sessions per week for 45 min per session) delivered as an inactive attention placebo by a research assistant in groups of three to five. The materials used in the GHE sessions will include audio-visual presentations, demonstrations, video clips, mini-games, oral quizzes, and posters and pamphlets on various health topics. The GHE sessions will be designed to raise awareness of general health issues and increase general health knowledge among an elderly population. The details of the GHE sessions are summarized in Table 3.

**Table 3:** Weekly topics of general health education sessions

Week	Topic	Content	Materials
1	Home safety	Strategies for removing potential home hazards to prevent residential accidents, such as the proper placement of sharp objects, the safe use of electric appliances, and fire safety.	• Audio-visual presentation • Pamphlets • Video clips
2	Choice of healthy foods	Information on food labels and allergies will be provided to facilitate the choice of healthy foods.	• Audio-visual presentation • Poster • Pamphlets • Mini-games • Oral quiz
3	Diet	Tips on healthy diet, such as a food pyramid and healthy recipes, will be introduced to establish a healthy eating style.	• Audio-visual presentation • Pamphlets • Oral quiz
4	Brain health	Concepts of the mind and memory will be introduced and mini-games relating to brain health will be played to raise awareness of the importance of maintaining brain health.	• Audio-visual presentation • Mini-games • Video clips
5	Hand care	The importance of hand and wrist care will be emphasized and the appropriate choice and use of hand-care products introduced.	• Audio-visual presentation • Demonstration
6	Foot care	The importance of foot and ankle care will be introduced, followed by information on maintaining foot and ankle care.	• Audio-visual presentation • Video clips
7	Flu prevention	Health information, including the symptoms, prevention, and treatment of flu, will be provided and ways to prevent flu discussed.	• Audio-visual presentation • Pamphlets
8	Handicrafts	The importance of developing hobbies and leisure activities will be discussed, followed by a demonstration of some common handicrafts.	• Audio-visual presentation • Demonstration • Mini-games

### Safety and adverse events

CBT is a clinically proven therapeutic intervention with no known associated risks. However, as one of the aims of CBT interventions is to promote independence, the participants will be instructed to increase their ADL, physical exercise, and social participation. Potential hazards will be discussed in the CBT sessions before these behavioral changes are effected. Information on safety precautions will be provided, and the participants will be aided in the development of strategies to minimize potential hazards and ensure safety. The therapists and research personnel will report any and all adverse events to the Departmental Research Committee of the Hong Kong Polytechnic University.

### Outcome measures

#### Primary outcome measure

##### Balance confidence

Our primary outcome of interest is FoF, which will be measured using the Chinese version of the Activities-specific Balance Confidence Scale (ABC) [34]. The ABC-C consists of 16 items representing specific situations in daily life rated on a scale from 0% (no confidence) to 100% (complete confidence). The ABC has been validated for use with community-dwelling elderly [42] and people with various medical conditions, such as Parkinson’s disease [43] and stroke [44, 45]. The ABC has also been translated into Chinese (Cantonese), and shows an excellent internal consistency (Cronbach’s alpha = .97) and a high test-retest reliability (intraclass correlation coefficient = .99) [34].

#### Secondary outcome measures

##### Fear-avoidance behavior

The participants’ engagement in fear-avoidance behavior will be assessed using the Chinese version of the Survey of Activities and Fear of Falling (SAFFE-C) [46]. The SAFFE-C is a self-reported inventory designed to measure the restriction on respondents’ activity created by FoF. The SAFFE-C consists of 22 items measuring the extent of individuals’ worry over performing 22 activities representing ADLs, mobility and social activity on a four-point Likert scale (0 = not at all worried, 1 = a little worried, 2 = somewhat worried, and 3 = very worried). The Chinese translation of the SAFFE shows excellent internal consistency (Cronbach’s alpha = .95) [46].

##### Balance

Balance ability will be measured using the Berg Balance Scale (BBS) [47], which is considered a valid measure of functional balance in various populations, such as stroke survivors and healthy older adults [48]. The BBS consists of 14 items, each rated on a five-point scale. A score of 41–56 indicates the ability to walk independently, 21–40 indicates the ability to walk with assistance, and 0–20 indicates wheelchair-bound movement.

##### Fall risk

Fall risk will be quantified using the Short-form Physiological Profile Assessment (S-PPA), which consists of five tests: a vision test, a proprioception test, a lower extremity muscle force test, a hand reaction time test, and a balance test [49]. Composite scores are measured on a seven-point scale according to the participants’ responses to the tests. Potential fall risk ranges from −2, representing a very low fall risk, to 4, which represents a very marked fall risk. The S-PPA has been shown to distinguish effectively recurrent fallers from non-fallers among community-dwelling older adults [50].

##### ADL

The respondents’ engagement in ADL will be measured using the Chinese version of the Lawton Instrumental Activities of Daily Living Scale (IADL-C) [51]. The scale’s nine items reflect the respondents’ level of independence in performing nine instrumental ADL: making telephone calls, using transportation, shopping, cooking, housekeeping, undertaking household repairs, doing the laundry, self-medicating, and handling finances.

##### Community reintegration

Community reintegration will be measured using the Chinese version of the Community Integration Measure (CIM-C) [52]. The CIM-C consists of ten items on a five-point scale representing the respondents’ self-reported sense of community reintegration. The CIM has been used for patients with various chronic illnesses, such as acquired brain injury [53] and stroke [52].

##### Health-related quality of life

Quality of life will be assessed using the Chinese version of the Short Form General Health Questionnaire (SF36-C) [54]. The SF36 consists of self-reported items related to physical functioning, role limitations due to physical health problems, bodily pain, general health, vitality, social functioning, and emotional well-being. Two summary scores obtained for a physical component scale (PCS) and a mental component scale (MCS) are converted into a score on a scale from 0 to 100, representing a continuum of disability in which scores of 0 and 100 refer to the maximum and minimum levels of disability, respectively.

### Data analysis

The data will be double-entered to enable validation. Simple descriptive statistics will be used to summarize the sociodemographic characteristics of the participants and other variables of interest. The normality of the data will be examined by a Kolmogorov–Smirnov test. Between-group comparisons at baseline will be performed using *t* tests, Kruskal–Wallis tests, chi-square tests, or Fisher’s exact test, as appropriate.

To measure the changes over time in variables of interest between the two study arms, mixed-effects models with adjustments for potential confounding variables, such as sociodemographic characteristics, will be used. Mixed-effects models go beyond the customary linear framework by incorporating random effects relating to participants. They account well for intra-correlated repeated measures data and accommodate missing data caused by dropouts, as long as the data are missing at random. Pearson and Spearman’s correlation tests, as appropriate, will be used to investigate the correlations between outcome variables. SPSS 17.0 will be used for the remaining statistical analysis, with a 5% level of confidence (two-sided) accepted for significance.

### Sample size

The sample size has been calculated using G*Power version 3.1.0, with an alpha level of 0.05 (one-tailed) and a study power of 80%. As no previous studies have addressed the effects of CBT in reducing FoF in stroke populations, the effect size used to calculate the sample size is the same as that calculated for our pilot sample of ten subjects (0.26), in which five subjects received the CBT, another five subjects received the GHE, and both groups received 45 min of TOBT. The ABC-C [34] is the primary outcome measure at eight weeks after the end of treatment. The required sample will, thus, comprise 76 subjects, with 38 per group. With reference to previous clinical trials [30, 40], we expect the dropout rate to be about 15%, requiring an extra six subjects per group to be recruited. Therefore, the planned sample size is 88.

## Discussion

FoF and actual falls create a vicious cycle with devastating consequences for patients with chronic stroke. In the community under study, more than half of the patients with stroke experience impaired subjective balance confidence and have suffered at least one fall since discharge. Interventions for fall prevention have focused on balance training, leaving the FoF of stroke survivors under-addressed. The aim of the proposed clinical trial is to evaluate the effectiveness of a combination of TOBT and CBT in reducing FoF, and in turn reducing fear-avoidance behavior, increasing balance, enhancing engagement in ADL, decreasing fall risk, promoting community reintegration, and enhancing the quality of life of patients with chronic stroke.

CBT is a form of clinically proven psychotherapeutic intervention designed to shape patients’ thinking and actions to achieve therapeutic goals. Research has shown that multidimensional programs with CBT components provide an effective means of treating FoF and reducing the incidence of falling among healthy older adults. This sheds light on the effects on stroke recovery of CBT combined with customary physical training. The inclusion of CBT with customary physical training will help to break the vicious cycle of FoF and actual falls and thus, enhance the rehabilitative outcomes of patients with chronic stroke.

One of the limitations of this study is not collecting data on actual falls. However, the main purpose of this study is to evaluate the augmenting effects of CBT on existing physiotherapy in enhancing subjective balance confidence. The occurrence of post-stroke falls is a complex issue involving the interplay between physical, psychological, behavioral, and environmental factors. Future studies could further examine the roles of subjective balance confidence in developing fall prevention strategies for patients with stroke. Besides, this study may involve community-dwelling stroke patients with a range of balance ability levels. Thus, the use of BBS may not be adequately sensitive to capture the balance improvement among subjects with mildly affected balance ability.

It is hoped that the results of this study will provide scientific evidence supporting the use of CBT to augment the effects of physiotherapy in enhancing subjective balance confidence and thus, stroke rehabilitation. If effective, our intervention will offer a safe, cost-effective, and readily transferrable therapeutic approach to clinical practice that reduces fear-avoidance behaviors and fall risk, improves balance and level of independence, enhances health-related quality of life, and decreases associated healthcare costs.

### Trial status

Recruitment started in October 2016. We target to complete recruitment during 2017.

## Abbreviations

ABC-C: Chinese version of the Activities-specific Balance Confidence Scale
ADL: Activities of daily living
BBS: Berg Balance Scale
CBT: Cognitive behavioral therapy
CIM-C: Chinese version of the Community Integration Measure
FoF: Fear of falling
GHE: General health education
IADL-C: Lawton Instrumental Activities of Daily Living (Chinese version)
RCT: Randomized controlled trial
SAFFE-C: Chinese version of the Survey of Activities and Fear of Falling in the Elderly
SF36-C MCS: Mental component of the Chinese version of the Short Form General Health Questionnaire
SF36-C PCS: Physical component of the Chinese version of the Short Form General Health Questionnaire
S-PPA: Short-form Physiological Profile Assessment
TOBT: Task-oriented balance training
